# Symptoms and Conditions Following the Subsidence of the Alcoholic Disease

**Published:** 1911-03

**Authors:** T. D. Crothers

**Affiliations:** Superintendent Walnut Lodge Hospital, Hartford, Conn.


### TEXAS MEDICAL JOURNAL.

Established July, 1885

F. E. DANIEL, M. D., - - - - - - Editor, Publisher and Proprietor

Published Monthly.—Subscription $1.00 a Year.

Vol. XXVI AUSTIN, MARCH, 1911. No. 9

The publisher is not responsible for the views of the contributors.

### Original Articles.

For Texas Medical Journal.

### Symptoms and Conditions Following the Subsidence of the Alcoholic Disease.

BY T. D. CROTHERS, M. D., SUPERINTENDENT WALNUT LODGE HOSPITAL, HARTFORD, CONN.

The degenerations that follow from syphilis may break out any time in later life and assume very strange forms. In puzzling cases, the fact of having had syphilis at one time is a clue to successful treatment, and in some Eastern cities prominent clinicians advise the use of mercury when in doubt.

The subsidence of the acute symptoms and the recovery of the case sustains this haphazard prescription. Experience shows that mercury in all cases with a syphilitic history is very nearly a specific remedy.

Recent very careful studies have indicated that an alcoholic history is not unfrequently followed in later life with very obscure puzzling symptoms which are traceable to some degeneration and injury due to alcoholic poisoning. Surgeons find it very practical to inquire for an alcoholic history before the operation. If this is clear, the operation and the prognosis and often the diagnosis will vary materially.

It is found that diseases of the respiratory organs are decidedly more fatal and less amenable to drugs, in those who have used alcohol than others, although the alcoholic addiction may have occurred years before. In many ways this fact appears that alcohol and drug taking has impressed the organism in some peculiar way, leaving it more susceptible to traumas and inflammations and with less ability to resist the various strains for repair.

One of the most practical phases of this question is the discovery of concealed addictions to alcohol and opium in persons who present very puzzling symptoms that seem to be unexplainable by ordinary diagnosis.

Where the patient acknowledges to have used alcohol or opium at some previous time, it is wise to infer that such use may be an influential factor in the present condition. Where these cases occur in ordinary business life, it is seldom difficult to follow up the line of cause and effect, and, while the patient may deny it, the circumstances indicate a continuous use, although in different forms of the same spirits and drugs, but when the cases are those in professional circles or among persons prominent in the business or political world, the effort to conceal the real facts is greater and more positive and the physician will be misled both consciously and unconsciously.

Thus a man who has used spirits to excess, meaning by this to have drank steadily and frequently been intoxicated, then becomes ostensibly abstinent, and lives many years as temperate and normal, suddenly becomes an invalid. His symptoms suggest grave disturbances that are unusual, unexplainable and are remarkable for their variability. The physician in despair of any clearer diagnosis will call it neurotic, neurasthenic and such other general names, and yet he is not able to determine the exact causes.

A great variety of remedies are suggested, including travel, diet and possibly wine and other remedies. The conditions become worse, and consultations only add confusion. Finally a concealed addiction is discovered.

If the patient is taking morphia and the remedy given by the physician contains morphia, a shrewd comparison of the effects will suggest the real cause. If wine or beer or any form of alcohol constitute a part of the remedy, the effects will very often indicate whether the remedy is a cause of the condition. An example is that of a person occupying a responsible position in social life who is known to be a spirit drinker, perhaps not to any excess, according to the so-called standards, at least not to have reached a toxic stage that has attracted attention. Such a person having obscure dietetic and neurotic symptoms should always be recognized as suffering from a possible spirit and drug degeneration.

This will usually be confirmed by a careful critical study. It not unfrequently happens that a new physician is called to treat some puzzling symptoms which the family doctor has failed to help. Should a strong tincture or a preparation of wine or beer be recommended, and the patient immediately recover from many of his distressing symptoms, a hint of previous use of it must be recognized.

These means after a while will lose their effects and other more complicating symptoms will appear and another physician sought for, who may repeat the same things, in effect using different drugs having the same spirit basis. Later, more obscure symptoms appear and the patient goes to the hospital and possibly dies. The varied treatment which he has received has all been injurious and has literally increased the difficulty, which was the concealed use of drugs and spirits, and was unrecognized by the physician.

A case of much prominence and some newspaper discussion was that of a lawyer who drank from early life, in so-called moderation. He was never seen intoxicated or appeared to vary much in emotion or mentality. At about 50 years of age he began to decline rapidly, and had pronounced symptoms of general dementia. Both body and mind were depressed and enfeebled. Many physicians were called and a great variety of remedies were used. No one suggested the removal of spirits, or that spirits was in any way responsible for the condition. His own impression encouraged by his medical advisers was that the removal of spirits would be fatal; that he had depended upon them so long they must be continued. Finally acute Bright's disease came on and he died.

Another man in the same neighborhood who had drank beer for years was threatened with the same exhaustion and dementia and was sent off to the mountains. Here the family physician insisted that all stimulants should be dropped. This was contrary to the patient's wish and to previous medical advice, and yet it was persisted in. The result was recovery, and the patient went back to business and lived many years.

These two cases represent types that are not uncommon, but are exceedingly significant, and indicate possibilities that ought to be recognized. Patients who come to physicians with a history of using proprietary drugs, which in most cases contain spirits and narcotics, should be the subject of very careful scrutiny, particularly to determine a history of the previous use of spirits.

With these facts, the diagnosis and treatment will be different and more rational. In these days of stress and strain, the use of alcohol, both socially and medicinally, is more or less common; also, its use in proprietary drugs and other remedies should be considered in every clinical study.

The old time delusion that alcohol has brought some stimulus or tonic properties to the body, has no place in modern research. It is now established beyond question that alcohol is an anesthetic, and as such may be very fascinating to some persons, particularly neurotics and worn-out people.

On all persons the continuous anesthesia of the nerve centers and cells produce pronounced effects of both degeneration and paralysis that in some way continues far down into the future, and always leaves a susceptibility which must be recognized in future diseases and injuries.

There are many eminent authorities on this point, and some of the statistics are very startling. The sudden collapse of men in middle life who have apparently lived along rational lines is not unfrequently traceable to alcoholic injuries at the beginning of their career. Like syphilis, the perversions and injuries have permanently impressed the organism and destroyed its vitality so that after a certain time the energy is exhausted and the patient dies.

This is strikingly confirmed in the statistics of deaths from pneumonia, particularly in persons in middle life. It is variously estimated that from 60 to 80 per cent of such deaths have a history of alcoholic or drug addiction some time during their life, or immediately before death.

Cerebral hemorrhage occurring in middle life and arteriosclerosis both show the same startling histories of the use of alcohol and drugs. It may be assumed that spirits is a very active cause of these two conditions, and at all events it is a very prominent predisposing cause.

The alcoholic or inebriate is never the same physically, even after long years of abstinence. There is lowered vitality and feeble resisting power, which is very evident whenever occasion calls for some unusual efforts. It is this fact that should be recognized, particularly in obscure cases, in protracted fevers and in nerve defects.

The concealed use of opium or morphia should be suspected in persons who have previously used alcohol and abstained, particularly in persons who present a variety of complex symptoms of great extreme of excitement and depression. Physicians, nurses and persons who have had some training in the care of the sick present symptoms that suggest secret drugs of some kind, and particularly drugs by the needle.

In some sections of the country the unusual demand for hypodermic needles suggests their use secretly, and probably morphia is the most commonly used of the drugs. Many irregular or thoughtless physicians give morphia boldly, telling the patient what it is, and in this way cultivate an addiction which may be concealed for a long time.

Other drugs are not unfrequently used, and most remarkable effects are attributed to them, which, when examined, are found to depend on some unknown drug used in connection with them. A man who has previously been an alcohol addict and is supposed to have given it up and becomes an enthusiastic defender of some proprietary medicine or unknown drug, should be regarded with suspicion. The common inference is that he is still using the original spirits or drugs under the cloak or pretense of using some other substance.

Tincture of oats has often been used in this way. Repeated examinations by the most careful students have failed to show the extravagant virtues claimed for tincture of oats by certain persons. In the same way certain proprietary drugs which, from examination, are known to have little or no special action on the system are praised for effects which can not possibly come from them.

The reformed inebriate and drug taker who continues to use drugs both regular or irregular is open to the suspicion. Many of these persons show hysterical credulity in all sorts of means and measures of restoration, and it is this class that frequently appeal to physicians for help. If the facts are not clear concerning their former life, the physician is very likely to give harmful drugs and leave them worse or perhaps cause the original addiction to break out again.

Such patients are paranoiacs in many ways with a great variety of neurotic and changeable dietetic disturbances, and an irregular heart's action and high-tensioned arteries are common symptoms. When they are emotionally excited persons, hemorrhage and sclerosis are common. Acute inflammation of the lungs is another common result, coming on with or without any exposure.

These persons are called chronic invalids and where they are able to pay the physician for his services are a very interesting class. The diagnosis is always variable, and each new physician called will make out a new case, and so the vicious circle is continued.

If the physician is very wise, he will avoid narcotics and alcoholics and always give remedies that will in some way prevent these conditions or at least neutralize them. If the patient has become a confirmed drug taker, who is certain to go from one thing to another, the effort should be to keep him on drugs that have little possible injury to the system, and this requires psychical study and psychical treatment.

It is this particular delusion that keeps alive different schools of medicine. Thus the homeopaths give dilutions of milk and sugar with very minute directions and suggestions, and the patient is convinced of the effects, and for years may be kept in a degree of health by depending on these mental props.

Recently the osteopaths have come into this field, and finding dislocations of almost every part of the body proceed to treat them, and the patient believes that he receives permanent benefit. These are most commendable to the reformed inebriate and drug taker for their mental effects. They are preventive and bring about results that are practical.

The physician should do this with far greater skill than the irregular. He should realize the paranoiac tendencies and the degeneracies that have come from alcoholic toxemia, and treat such people with great satisfaction. As to the final cure, that is another matter. Most of them need help the rest of life.

To them it is drug help, material and tangible. To the physician it is mental and psychical, and drugs are only essential to keep up the impression. A practical case will bring out these facts clearly. A man who had drank for twenty years to great excess suddenly stopped, and in the course of a year a great variety of obscure symptoms, both rheumatic and neurotic, followed. His physician, who was aware of his previous life, after a very thorough examination, placed him on placebos and a particular diet, which was to be carried out with great minuteness. The results were not very satisfactory for the first six months, but the physician persisted giving him minute suggestions and urging hydropathic measures. A change took place, and a steady restoration followed. He became dependent on the physician, who encouraged this confidence in every possible way. A great variety of means and measures were used and the patient's mind was absorbed with the efforts to get well.

He was in active business, but the physician insisted on controlling his acts and conduct so that everything could be made to increase his vitality and physical health. His recovery at the end of four or five years was very complete. He is now occupying a responsible position, doing good work, but dependent largely on his physician, who is consulted on all matters, both business and social.

If the physician had not persistently followed up this case with both psychical and physical measures, the man would have fallen into the hands of quacks, relapsed and died, or if the physician had been careless and treated him with opium he would have gone back at once; or if tinctures had been given the results would have been fatal.

This case illustrates the possibilities of future work for physicians. Obscure cases with an alcoholic history ought always to attract the greatest of attention. The physician should exercise the highest psychical skill in determining the possible condition and the best remedies, or absence of remedies, that would prevent the body and mind from relapsing.

This is the unknown field that if occupied by the regular physician would diminish the work of quacks.

